# Molecular and proteomic insight into Notch1 characterization in hepatocellular carcinoma

**DOI:** 10.18632/oncotarget.9203

**Published:** 2016-05-06

**Authors:** Catia Giovannini, Manuela Minguzzi, Filippo Genovese, Michele Baglioni, Alessandra Gualandi, Matteo Ravaioli, Maddalena Milazzo, Simona Tavolari, Luigi Bolondi, Laura Gramantieri

**Affiliations:** ^1^ Center for Applied Biomedical Research (CRBA), S.Orsola-Malpighi University Hospital, Bologna, Italy; ^2^ Department of Medical and Surgical Sciences, University of Bologna, Bologna, Italy; ^3^ C.I.G.S., University of Modena and Reggio Emilia, Bologna, Italy; ^4^ Department of Medical and Surgical Sciences, General and Transplant Surgery Unit, University of Bologna, Bologna, Italy; ^5^ Department of Experimental Diagnostic Specialty Medicine, University of Bologna, Bologna, Italy

**Keywords:** Notch1, invasion, proteomic, markers, HCC

## Abstract

Hepatocellular carcinoma (HCC) ranks fifth in frequency worldwide amongst all human cancers causing one million deaths annually. Despite many promising treatment options, long-term prognosis remains dismal for the majority of patients who develop recurrence or present with advanced disease. Notch signaling is an evolutionarily conserved pathway crucial for the development and homeostasis of many organs including liver. Herein we showed that aberrant Notch1 is linked to HCC development, tumor recurrence and invasion, which might be mediated, at least in part, through the Notch1-E-Cadherin pathway. Collectively, these findings suggest that targeting Notch1 has important therapeutic value in hepatocellular carcinoma. In this regard, comparative analysis of the secretome of HepG2 and HepG2 Notch1 depleted cells identified novel secreted proteins related to Notch1 expression. Soluble E-Cadherin (sE-Cad) and Thrombospondin-1 (Thbs1) were further validated in human serum as potential biomarkers to predict response to Notch1 inhibitors for a tailored individualized therapy.

## INTRODUCTION

Hepatocellular carcinoma (HCC) is the third leading cause of cancer mortality worldwide with an increased incidence throughout the world [1]. Although the introduction of screening programs among high-risk populations leads to earlier diagnosis, the majority of patients present with intermediate or advanced-stage disease. Sorafenib (Nexavar) remains the only systemic therapy recognized to improve overall survival in patients with advanced HCC [2]. However, for patients who are intolerant of or progress on sorafenib no other drug has been approved as second line option [3]. Thus, the development of new agents to block HCC progression is the primary research objective. Notch pathway has been described to be involved in cell fate determination, cell differentiation, proliferation and death [4]. Notch promotes cell survival, angiogenesis and treatment resistance in numerous cancers, making it a promising target for cancer therapy [5, 6]. It also crosstalks with other critical oncogenes, providing a means to affect numerous signaling pathways with one intervention [5]. Most of the studies have focused on Notch1 which has been shown to be associated to invasion and metastasis in pancreatic cancer cells [7], in prostate cancer [8] and in melanoma [9]. Data regarding Notch1 involvement in HCC are limited and ambiguous in terms of anti-tumoral effects following its inhibition [10, 11], a discrepancy that may be due to the high context dependency of the Notch cascade [12]. We and Zhou reported abnormal accumulation of Notch1 in HCC compared to surrounding non tumor tissue [13, 14] raising the possibility that deregulation of Notch1 receptor may participate to HCC tumorigenesis and cancer progression.

Set of Notch inhibitors have been developed. Most of these inhibitors show anti-tumor effects in preclinical studies [15]. Anti-Notch2 treatment blocks the development of a broad range of tumors in a mouse model with primary liver cancer whereas the inhibition of Notch1 increases the development of cholangiocarcinoma like tumors [16]. However, environmental factors are also involved in routes to HCC development suggesting that Notch pathway activation should be analysed in different liver cancer models as previously discussed [16].

Blocking monoclonal antibodies (mAb) directed against Notch1 are under investigation [17]. However, serum biomarkers to predict response to Notch inhibitors are not available. Similarly, it is not always possible to obtain tumor tissue samples to test Notch1 expression in patients with HCC on cirrhosis who may display coagulation impairments or other contraindications. Therefore, the use of serum markers related to the expression of Notch1 could be of great help in clinical practice.

To this aim, we have performed a quantitative proteomic analysis of the culture media of HepG2 cells against Notch1 depleted cells using a gel free approach. We have identified secreted proteins involved in cytoskeleton organization, cell adhesion and regulation of cellular metabolic process that can be considered as sensitive indicators of Notch1 expression. Since among the secreted proteins were identified proteins involved in cell adhesion, we analysed whether Notch1 could play a role in the invasion of liver cancer cells. Interestingly, we found out that Notch1 regulates the EMT (Epithelial-mesenchymal-transition) process for which E-Cadherin is a well-established hallmark. We also found out that Notch1 regulates E-Cadherin expression both at transcriptional and post-transcriptional level and that their expression is beneficial for invasiveness. We also proved that Notch1 and E-Cadherin protein expression positively correlates in human HCCs and those patients with high Notch1 and E-Cadherin expression show shorter time to recurrence (TTR). Finally, we uncovered that the lack of Notch1 expression could inhibit tumor's development *in vivo* contributing to the “field effect”.

## RESULTS

### Quantitative proteomic analysis reveals changes of protein expression in media of Notch1 depleted cells compared to control cells

**Figure 1:**
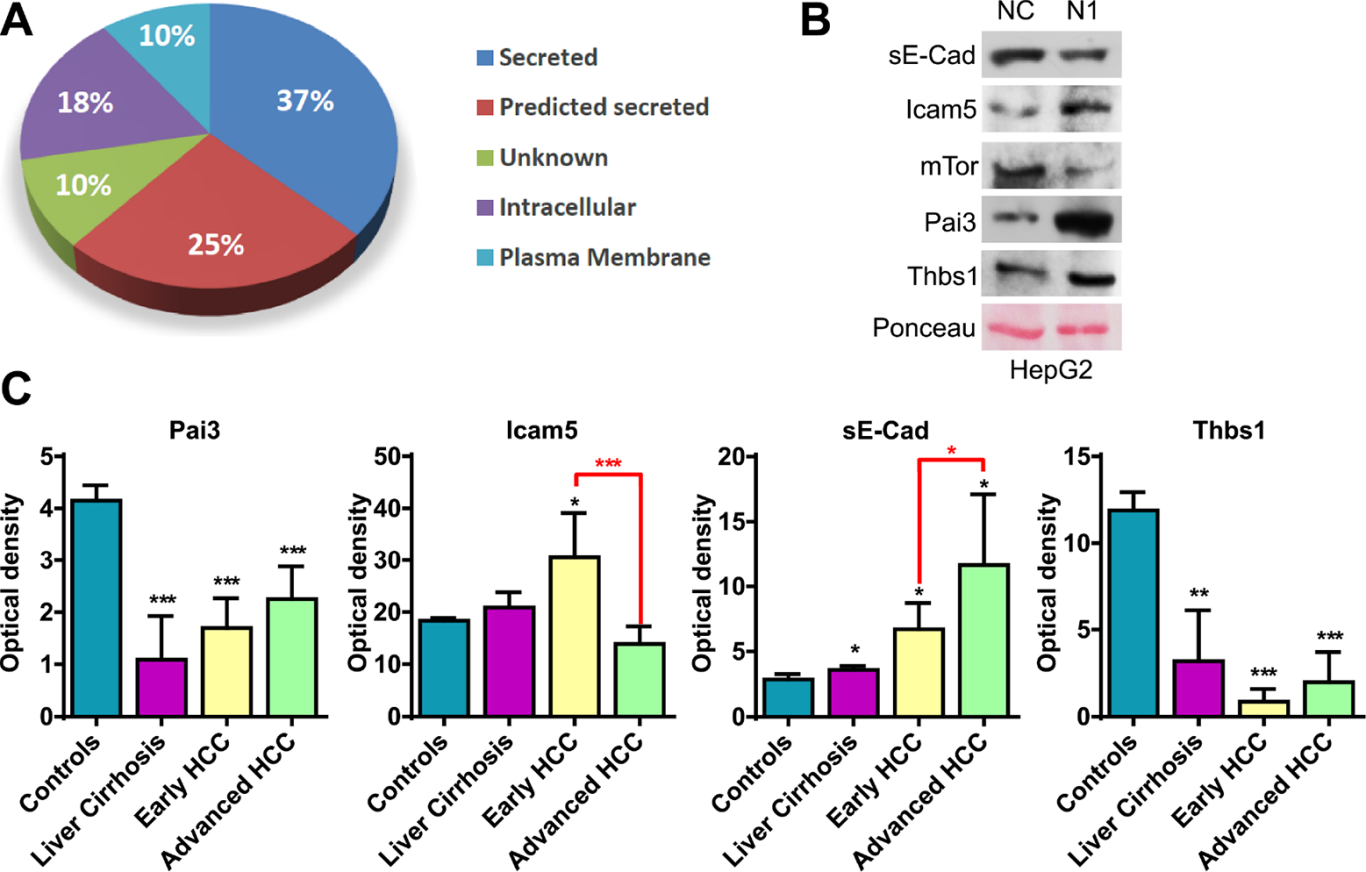
Analyses of secreted proteins (**A**) Subcellular localization of significantly altered proteins associated to Notch1 expression. (**B**) Western blot analysis confirms changes in protein expression in Notch1 depleted cells. Ponceau serves as proteins loading control. NC: negative control shRNA; N1: Notch1 shRNA. (**C**) Proteins found to be related to Notch1 expression in cell culture medium were analyzed in serum sample from patients with liver cirrhosis, early HCC, advanced HCC or healthy controls. Statistical analysis was performed by comparing patients to healthy controls (black asterisk) or by comparing early vs. advance HCCs (red asterisk) *P < 0.05; **P < 0.01; ***P < 0.001 (by two tailed student's *t* test).

Media from HepG2 control cells and Notch1 depleted cells were investigated for differences in secreted proteins that could be associated to Notch1 expression. Using a gel free proteomic approach and high-resolution mass spectrometry a total of 89 proteins were significantly altered (*P* < 0.05), with 37 proteins up-regulated and 52 down-regulated in Notch1 depleted cells. The list of up-regulated and down-regulated proteins is shown in Tables 1 and 2. According to SignalIP, secreted proteins represent 37% of the identified proteins. However to elucidate if proteins could use alternative secretion pathways, each protein was analyzed with SecretomeP. Remarkably 25% of proteins showed an NN-score of > 0.5, which predicts non signal peptide-triggered protein secretion and correlates with a non-classical protein secretion pathway. Among the 89 identified proteins, we also detected plasma membrane proteins (10%) and intracellular proteins (18%), presumably from dead cells (Figure 1A).

**Table 1:** List of up-regulated proteins in the conditioned media associated to Notch1

IPI Number	Gene Symbol	Protein name	N1/CTRL	Score
IPI00166060	LGI4	Isoform 1 of Leucine-rich repeat LGI family member 4	9999	0,9784
IPI00888255	LOC100291104	hypothetical protein XP_002346815	9999	0,9643
IPI00902507	ADAM5P	Putative disintegrin and metalloproteinase domain-containing protein 5	9999	0,968
IPI00304086	CTCFL	Transcriptional repressor CTCFL variant B1	9999	0,9537
IPI00184848	ABCB11	Bile salt export pump	9999	0,9653
IPI00026307	CDC40	Pre-mRNA-processing factor 17	9999	0,9701
IPI00016690	LATS2	Serine/threonine-protein kinase LATS2	9999	0,9878
IPI00853031	MRPL23	Uncharacterized protein	9999	0,9537
IPI00012048	NME2;NME1	Nucleoside diphosphate kinase A	9999	0,9745
IPI00026327	Not provided	134 kDa protein	9999	0,9529
IPI00025363	GFAP	Isoform 1 of Glial fibrillary acidic protein	9999	0,964
IPI00021146	PRPF18	Isoform 1 of Pre-mRNA-splicing factor 18	9999	0,9545
IPI00021727	C4BPA	C4b-binding protein alpha chain	10,997	0,9784
IPI00953689	AHSG	Alpha-2-HS-glycoprotein	10,776	1
IPI00554648	KRT8	Keratin, type II cytoskeletal 8	10,202	0,968
IPI00401829	C14orf38	Uncharacterized protein C14orf38	8,459	0,9701
IPI00844508	CGN	Cingulin	7,104	1
IPI00219563	PLCB1	Phosphatidylinositol-4,5-bisphosphate phosphodiesterase beta-1	6,449	0,964
IPI00002993	TAF9	Transcription initiation factor TFIID subunit 9	5,613	1
IPI00397016	FOXO6	Forkhead box protein O6	5,207	0,9784
IPI00012011	CFL1	Cofilin-1	4,808	0,9924
IPI00001895	PCDH8	Isoform 1 of Protocadherin-8	4,223	0,9548
IPI00220644	PKM2	Isoform M1 of Pyruvate kinase isozymes M1/M2	4,071	1
IPI00795830	AHSG	Uncharacterized protein	3,114	1
IPI00306322	COL4A2	Collagen alpha-2(IV) chain	3,092	0,968
IPI00290456	ICAM5	Intercellular adhesion molecule 5	2,928	0,9784
IPI00032258	C4A	Complement C4-A	2,821	1
IPI00027497	GPI	Glucose-6-phosphate isomerase	2,73	1
IPI00021754	44M2.3	Isoform 2 of Putative RNA exonuclease NEF-sp	2,728	0,9537
IPI00007221	SERPINA5	Plasma serine protease inhibitor	2,674	1
IPI00217963	KRT16	Keratin, type I cytoskeletal 16	2,577	0,968
IPI00005690	MATN3	Matrilin-3	2,498	0,9878
IPI00887739	Not provided	Uncharacterized protein	2,441	1
IPI00021885	FGA	Isoform 1 of Fibrinogen alpha chain	2,418	1
IPI00008787	NAGLU	Alpha-N-acetylglucosaminidase	2,286	0,9545
IPI00794873	Not provided	Uncharacterized protein	2,221	0,9956
IPI00026260	NME2;NME1	Isoform 1 of Nucleoside diphosphate kinase A	2,029	1

**Table 2:** List of Down-regulated proteins in the conditioned media associated to Notch1

IPI Number	Gene Symbol	Protein name	N1/CTRL	Score
IPI00514893	DAAM2	Disheveled-associated activator of morphogenesis 2	−9999	0,9537
IPI00644712	XRCC6	X-ray repair cross-complementing protein 6	−9999	0,9784
IPI00029723	FSTL1	Follistatin-related protein 1	−9999	0,9745
IPI00216298	TXN	Thioredoxin	−9999	1
IPI00006608	APP	Isoform APP770 of Amyloid beta A4 protein (Fragment)	−9999	1
IPI00021000	SPP1	Osteopontin isoform b precursor	−9999	1
IPI00168866	MDGA1	MAM domain-containing glycosylphosphatidylinositol anchor protein 1	−9999	0,9701
IPI00027547	DCD	Dermcidin	−9999	0,9722
IPI00290358	C16orf91	Chromosome 16 open reading frame 91	−9999	0,9653
IPI00003802	MAN2A1	Alpha-mannosidase 2	−9999	0,9537
IPI00164352	ZNF292	Isoform 1 of Zinc finger protein 292	−9999	0,9745
IPI00430472	ASCC3	Activating signal cointegrator 1 complex subunit 3	−9999	0,9797
IPI00297859	MLL2	Isoform 1 of Histone-lysine N-methyltransferase MLL2	−9999	0,9537
IPI00002804	PKN2	Serine/threonine-protein kinase N2	−9999	0,9722
IPI00007193	ANKRD26	Isoform 2 of Ankyrin repeat domain-containing protein 26	−9999	0,9745
IPI00220827	TMSB10	Thymosin beta-10	−9999	0,9701
IPI00412694	ANO5	Anoctamin-5	−9999	0,9797
IPI00142538	SETX	Isoform 1 of Probable helicase senataxin	−9999	0,9646
IPI00375578	SASS6	Spindle assembly abnormal protein 6 homolog	−9999	0,9722
IPI00414896	RNASET2	Isoform 1 of Ribonuclease T2	−9999	0,9784
IPI00021812	AHNAK	AHNAK Neuroblast differentiation-associated protein AHNAK	−9999	0,9745
IPI00741855	KRT39	KRT39 Keratin, type I cytoskeletal 39	−9999	0,9701
IPI00329679	ZWILCH	Isoform 1 of Protein zwilch homolog	−9999	1
IPI00556369	SMG1	SMG1 Isoform 3 of Serine/threonine-protein kinase SMG1	−9999	0,9653
IPI00401831	PLEKHH1	Pleckstrin homology domain-containing family H member 1	−9999	1
IPI00003818	KYNU	Kynureninase	−9999	0,9701
IPI00013400	MMP7	Matrilysin	−9999	0,9797
IPI00096066	SUCLG2	Succinyl-CoA ligase [GDP-forming] subunit beta, mitochondrial	−9999	0,9537
IPI00178352	FLNC	Isoform 1 of Filamin-C	−9999	0,964
IPI00791999	PSMD9	26S proteasome non-ATPase regulatory subunit 9	−9999	0,9722
IPI00879906	Not provided	92 kDa protein	−9999	0,9722
IPI00031410	MTOR	Serine/threonine-protein kinase mTOR	−9999	0,9722
IPI00302927	CCT4	complex protein 1 subunit delta	−9999	0,9529
IPI00217992	DST	Isoform 3 of Bullous pemphigoid antigen 1	0,023	0,95
IPI00291175	VCL	Isoform 1 of Vinculin	0,058	1
IPI00293878	CMTM8	CKLF-like MARVEL transmembrane domain-containing protein 8	0,06	0,9722
IPI00025252	PDIA3	Protein disulfide-isomerase A3	0,078	0,9797
IPI00219249	CNTNAP1	Contactin-associated protein 1	0,089	0,9722
IPI00789146	Not provided	13 kDa protein	0,103	0,9722
IPI00847471	IGSF9B	IGSF9B Protein turtle homolog B	0,145	0,9722
IPI00883591	ZNHIT1	ZNHIT1 Conserved hypothetical protein	0,17	0,9722
IPI00009852	ATP6V0A4	Proton ATPase 116 kDa subunit a isoform 4	0,234	0,9537
IPI00180408	MYH15	Myosin-15	0,271	1
IPI00296099	THBS1	THBS1 Thrombospondin-1	0,283	1
IPI00935923	LOC100286949	hypothetical protein XP_002342730	0,303	0,9548
IPI00072377	SET	Isoform 1 of Protein SET	0,314	0,9924
IPI00218319	TPM3	Isoform 2 of Tropomyosin alpha-3 chain	0,317	1
IPI00400826	CLU	Isoform 1 of Clusterin	0,399	1
IPI00013256	CSTF2	Isoform 1 of Cleavage stimulation factor subunit 2	0,411	0,9537
IPI00385149	PTMA	Putative uncharacterized protein	0,412	1
IPI00103381	IPCEF1	Isoform 2 of Interactor protein for cytohesin exchange factors 1	0,433	0,964
IPI00008529	RPLP2	60S acidic ribosomal protein P2	0,49	1

### Biological function and pathway analysis for deregulated proteins

To identify altered biological functions that might be associated to Notch1 expression, secreted proteins were classified using DAVID functional annotation tool (Table 3). Cytoskeleton organization, cell adhesion and regulation of cellular protein metabolic process were among the top altered functions with *p* values ranging from 2.1E–05 to 3.3E–05.

**Table 3:** Biological function

Gene Symbol	*P* Value
**Regulation of organelle organization**	9,80E–06
CTCFL	
SET	
CFL1	
DST	
MTOR	
TMSB10	
**Cytoskeleton organization**	2,10E−05
CFL1	
DAAM2	
DST	
KRT16	
KRT8	
SASS6	
TMSB10	
**Cell adhesion**	2,80E−05
APP	
CNTNAP1	
DST	
ICAM5	
NME1−NME2	
PCDH8	
THBS1	
VCL	
**Regulation of cellular metabolic process**	3,30E−05
CTCFL	
SET	
TAF9	
APP	
MTOR	
SERPINA5	
THBS1	
**Membrane Organization**	1,40E−04
AHSG	
APP	
ICAM5	
NME1−NME2	
SERPINA5	
THBS1	
**Endocytosis**	2,20E−04
AHSG	
APP	
ICAM5	
NME1−NME2	
THBS1	
**Regulation of cellular component size**	4,80E−04
TAF9	
APP	
CFL1	
MTOR	
TMSB10	
SPP1	
**Response to organic substance**	2,60E−03
LATS	
TAF9	
AHSG	
CFL1	
KYNU	
MTOR	
NME1−NME2	
SPP1	
THBS1	
**Vesicle mediated transport**	7,60E−03
AHSG	
APP	
ICAM5	
NME1−NME2	
THBS1	
**Cell death**	2,30E−02
TAF9	
CLU	
CFL1	
NME1−NME2	
THBS1	
**Phospate metabolic process**	4,30E−02
LATS2	
SMG1	
APP	
CFL1	
MTOR	
NME1	
PKN2	
THBS1	

### Western blot confirms that key protein expression changes in Notch1 depleted cells

Global quantitative proteomic analysis identified the expression changes of a large number of proteins in Notch1 depleted cells. To confirm that some of these expressions change, we selected several proteins for validation by immunoblotting including Serpinb5 (Pai3), Icam5, Thrombospondin-1 (Thbs1) whose roles are well established in cancer. Remarkably, these proteins are involved in all the significantly altered pathways (Table 3). mTor was used as a control because it has been described as a Notch1 target [18]. Soluble E-Cadherin (sE-Cad) was not detected in proteomic analysis, presumably due to a non-efficient protein extraction. Given the high relevance of sE-Cad to tumor progression [19], it was also assayed by western blot. As shown in Figure 1B cleaved sE-Cad expression was down-regulated in Notch1 depleted cells. Increased expressions of Pai3 and Icam5 and decreased expression of mTor were also validated.

Conversely, western blot results did not confirm the MS quantification data for Thbs1 that results more expressed in Notch1 depleted cells compared to negative control. In human, five genes with strong homology encoding Thbs1 through Thbs5 have been identified [20]. Probably, the high homology of THBS genes makes it difficult to distinguish them by mass spectrometry, which explains the opposite results obtained by western blot and MS.

### Thbs1 and sE-Cad are candidate biomarkers to discriminate patients with HCC from controls

One of the goals of this study was to identify serum biomarkers of propensity towards tumor response to Notch1 inhibitors as well as of indicators of treatment efficacy. Proteins found to be related to Notch1 expression in cell culture medium were analyzed in serum of patients with cirrhosis, early HCC, advanced HCC or healthy controls (Figure 1C). All the analyzed proteins have been identified in serum. Among the deregulated proteins Pai3, sE-Cad and Thbs1 significantly discriminated patients from controls whereas sE-Cad and Icam5 showed the ability to discriminate early from advanced HCCs (Figure 1C). Thbs1, sE-Cad and Icam5 deserve attention in future studies as non-invasive biomarkers of response to Notch1 inhibitors.

### Thbs1 serum levels negatively correlates with Notch1 expression in human HCC

Notch1 expression was evaluated by immunohistochemistry in 18 early HCCs of which we also had serum. A significant inverse correlation was determined between Notch1 and Thbs1 serum levels evaluated by ELISA (Pearson's test: *p* < 0.05) (Supplementary Figure 1) confirming a role of Notch1 in the regulation of Thbs1.

### Targeting Notch1 decreases HCC cells invasion *in vitro*

To clarify the role of Notch1 in the context of HCC we employed HepG2, SNU398 and SNU449 cells as *in vitro* models. The nuclear localization of NICD (Notch Intracellular Domain) suggests the activation of the receptor in the analyzed cell lines (Supplementary Figure 2A). According to this last remark, the expression of HES1 and CYCLIN D1 target genes is down- regulated in response to Notch1 stable silencing (Supplementary Figure 2B).

**Figure 2:**
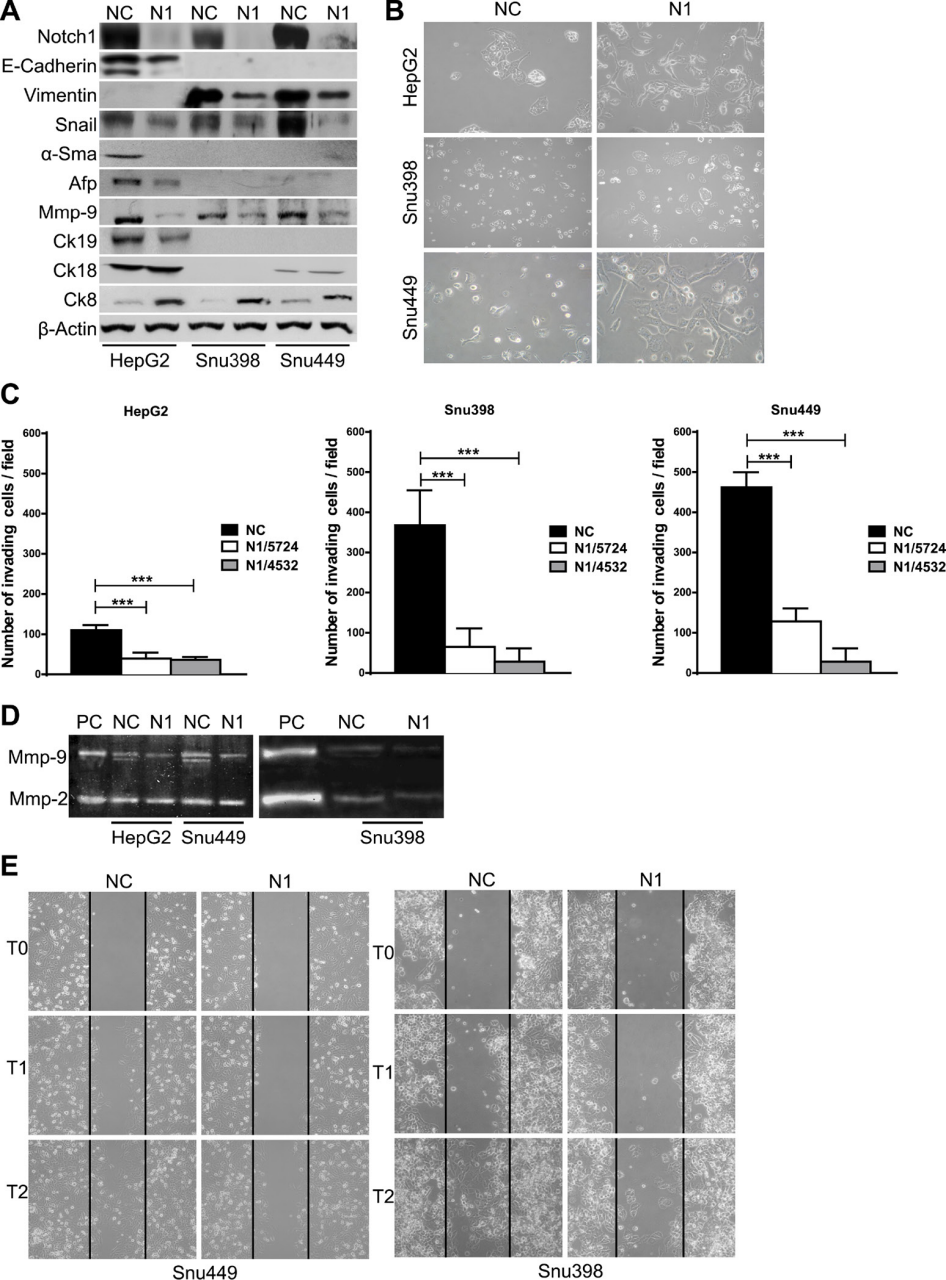
Effect of Notch1 knockdown (**A**) Proteins expression analysis in negative control cells and in Notch1 silenced cells by western blot. (**B**) Morphology of negative control shRNA and Notch1 depleted cells. (**C**) Difference in invasiveness ability of negative control cells and Notch1 silenced cells. ShOligos targeted to different Notch1 exons were used (N1/5724 and N1/4532). Results are the mean of three independent experiments (+/− S.E.). ***P < 0.001 (by two tailed student's *t* test). (**D**) Zymographic analysis of the Mmp-9 and Mmp-2 activity. PC: positive control. (**E**) Wound healing assay demonstrates that there are significantly more negative control cells that migrate into the wound area compared with Notch1 silenced cells. NC: negative control shRNA; N1: Notch1 shRNA.

**Figure 3:**
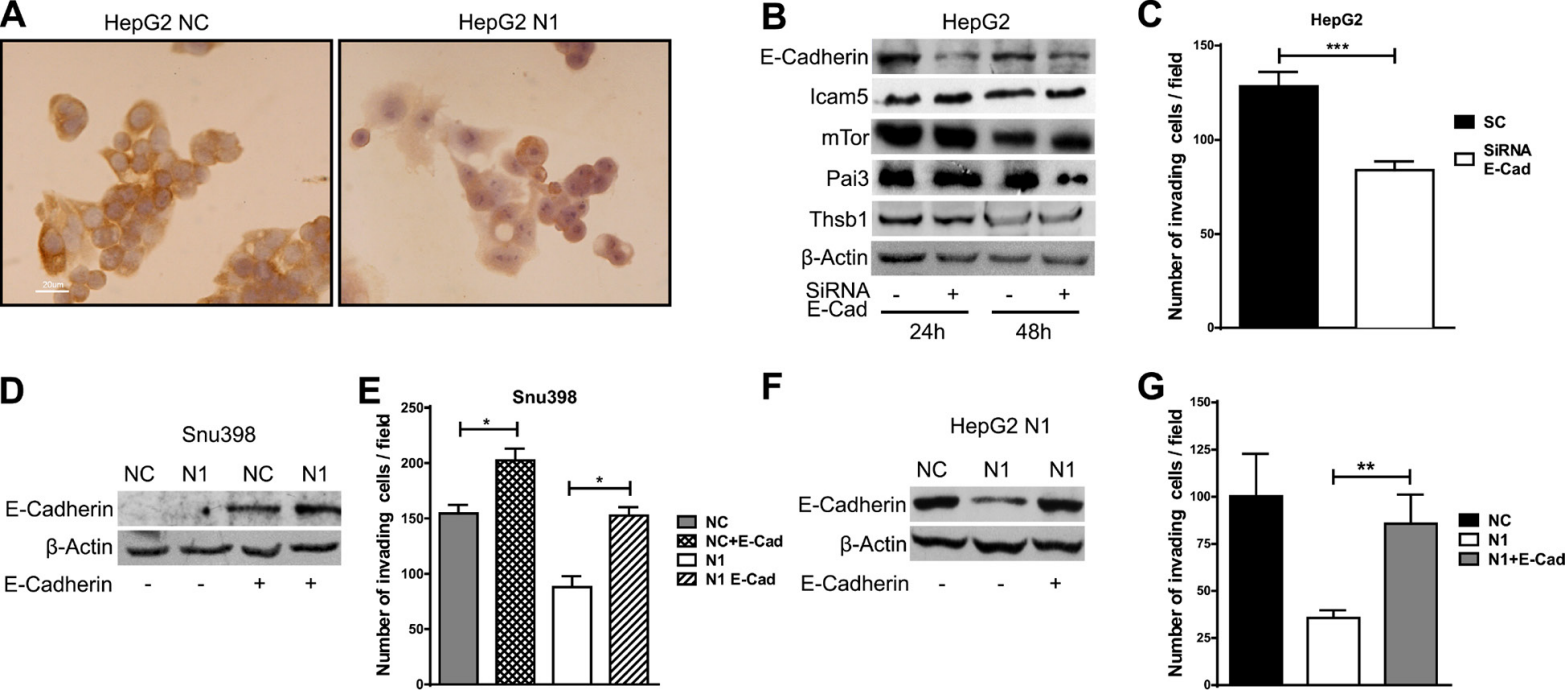
E-Cadherin induces invasion in hepatocellular carcinoma (**A**) Expression and subcellular localization of E-Cadherin, as detected by the immunoperoxidase method in HepG2 cell line. Positive staining was observed in cell membranes. Nuclei were counterstained with hematoxylin. Original magnification 40X. NC: negative control shRNA; N1: Notch1 shRNA. (**B**) HepG2 cells were transfected with E-Cadherin siRNA (E-Cad) or scrambled RNA and proteins expression analysis was evaluated 24 h and 48 h post-transfection by western blot. (**C**) Difference in invasiveness ability of HepG2 control cells and E-Cad silenced cells evaluated 48 h post-transfection. Results are the mean of three independent experiments (+/− S.E.). ***P < 0.001 (by two tailed student's *t* test). (**D**–**G**) SNU398 and HepG2 cells stable silenced for Notch1 (N1) and negative control (NC) were transfected with E-Cadherin expressing vector or with empty vector and difference in invasiveness ability was evaluated. Results are the mean of three independent experiments (+/− S.E.) *P < 0.05; *P < 0.01 (by two tailed student's *t* test).

Compared with control cells Notch1 KD cells underwent significant morphologic changes, which included a larger, flattened phenotype and tighter, more numerous cell-cell contacts (Figure 2B). Moreover, Notch1 silenced cells showed a low level of penetration through the matrigel-coated membrane, decreased Mmp-9 activity and reduced capacity to migrate into the wound area compared with the NC–infected cells (Figure 2C–2E). Additionally numerous mesenchymal related proteins including Keratin 19 (Ck19), Vimentin, Snail, Alpha-Sma and Mmp-9 were significantly down-regulated whereas epithelial markers including Keratin 8 and E-Cadherin resulted up-regulated and down-regulated respectively (Figure 2A). To confirm an E-Cadherin down-regulation in N1 silenced HepG2 cells we performed immunocytochemistry. As shown in Figure 3A NC cells demonstrated a more abundant E-Cadherin expression with a cell surface pattern than Notch1 depleted cells. Conversely, semi-quantitative RT-PCR analysis revealed an increased expression of E-Cadherin in N1 silenced cells compared to negative control (Supplementary Figure 2B) in line with Snail reduction suggesting that Notch1 regulates E-Cadherin levels in HCC by transcriptional and post-transcriptional mechanisms. Gene expression of N-CADHERIN, SNAIL, TWIST and VIMENTIN were also analysed. No difference was observed for all these genes following Notch1 down-regulation (Supplementary Figure 2B). We previously showed that Notch1 down-regulation does not affect cell viability but reduces cell proliferation [21]. To rule out that the inhibitory effects of Notch1 down-regulation on cell migration were not a consequence of reduced cell growth, the expression of the proliferation marker Ki-67 was analysed on wound-healing assay. The analysis was performed on SNU449 cells that are highly proliferating and have a high ability to penetrate through the matrigel-coated membrane. In the wound area we found both positive and negative cells for Ki67 protein expression suggesting that migration is not affected by cell proliferation (Supplementary Figure 3A).

### Notch1 and E-Cadherin contribute to the invasion of HCC

To determine if the reduced E-Cadherin levels were functionally associated with lower capacity to invade of Notch1 KD cells, HepG2 cells were transfected with human E-Cadherin siRNA (Figure 3B) and matrigel invasion assay was used to examine the invasive potential of E-Cadherin silenced cells. As illustrated in Figure 3C, E-Cadherin siRNA transfected cells showed a low level of penetration through the matrigel-coated membrane compared with cells transfected with the control siRNA. Interestingly, secreted proteins regulated by Notch1 were not affected by E-Cadherin inhibition with the exception of Pai3 suggesting that Notch1 and E-Cadherin (Figure 3B) regulate different pathways.

To further confirm the role of E-Cadherin in cell invasion, E-Cadherin was over-expressed in SNU398 Notch1 KD and in NC control cells (Figure 3D). E-Cadherin expression resulted in higher penetration of NC cells through the matrigel-coated membrane compared with the control pcmv-transfected cells (Figure 3E). Overexpression of E-Cadherin reverted effects of Notch1 ablation on cell motility in SNU398 cells. The same result was obtained in HepG2 Notch1 depleted cells following the re-expression of E-Cadherin. Our data shows an additive effect of Notch1 and E-Cadherin on invasion meaning the cells that do not express E-Cadherin take more advantage from Notch1 silencing. Indeed reduced cell migration was more evident in SNU398 and SNU449 than in HepG2 cells upon Notch1 silencing (Figure 2C). In agreement with this observation, several recent studies describe E-Cadherin re-expression in advanced metastatic tumors [22]. Furthermore, knockdown of E-Cadherin in the Notch depleted mammalian intestinal stem cells tumor resulted in strong suppression of cell mass formation [23].

### Notch1 and E-Cadherin protein levels correlate in human HCC

To assess whether our *in-vitro* findings reflect the biology of human HCC, Notch1 and E-Cadherin protein expression were analysed in 38 surgically resected HCCs by western blots.

**Figure 4:**
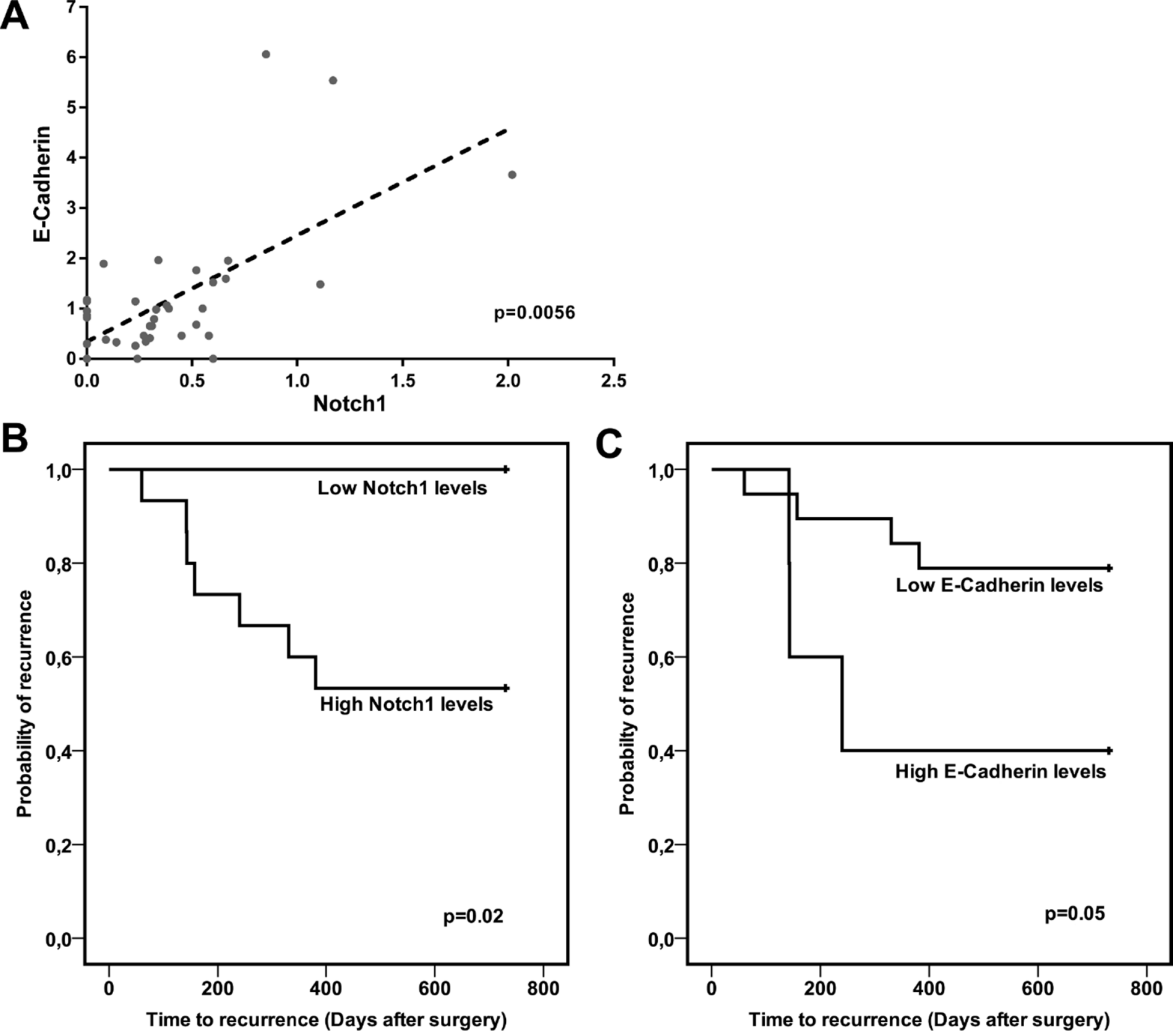
Notch1 and E-Cadherin correlate in human HCC and predict TTR (**A**) Scatter plot showing the relationship between Notch1 and E-Cadherin protein expression in 38 studied human HCCs. *P* = 0.0056 (by two tailed student's *t* test). (**B–C**) Association between Notch1 and E-Cadherin levels and TTR of surgically resected HCC patients. High and low, Notch1 and E-Cadherin expressions were categorized according to the mean value. Data on TTR were missing in 14 cases. Log-rank *P* values were from Kaplan-Meier analysis.

A significant positive correlation between Notch1 and E-Cadherin proteins accumulation was found (Pearson's test: *p* < 0.01) (Figure 4A). HCC_s_ were dichotomized based on Notch1 and E-Cadherin expression and the cut-off values were chosen based on median values in the whole series of HCC tissues. Both high Notch1 and E-Cadherin levels in HCC tissue were associated with shorter time to recurrence (TTR) (Figure 4B–4C).

### Notch1 expression in liver is associated with HCC development

**Figure 5:**
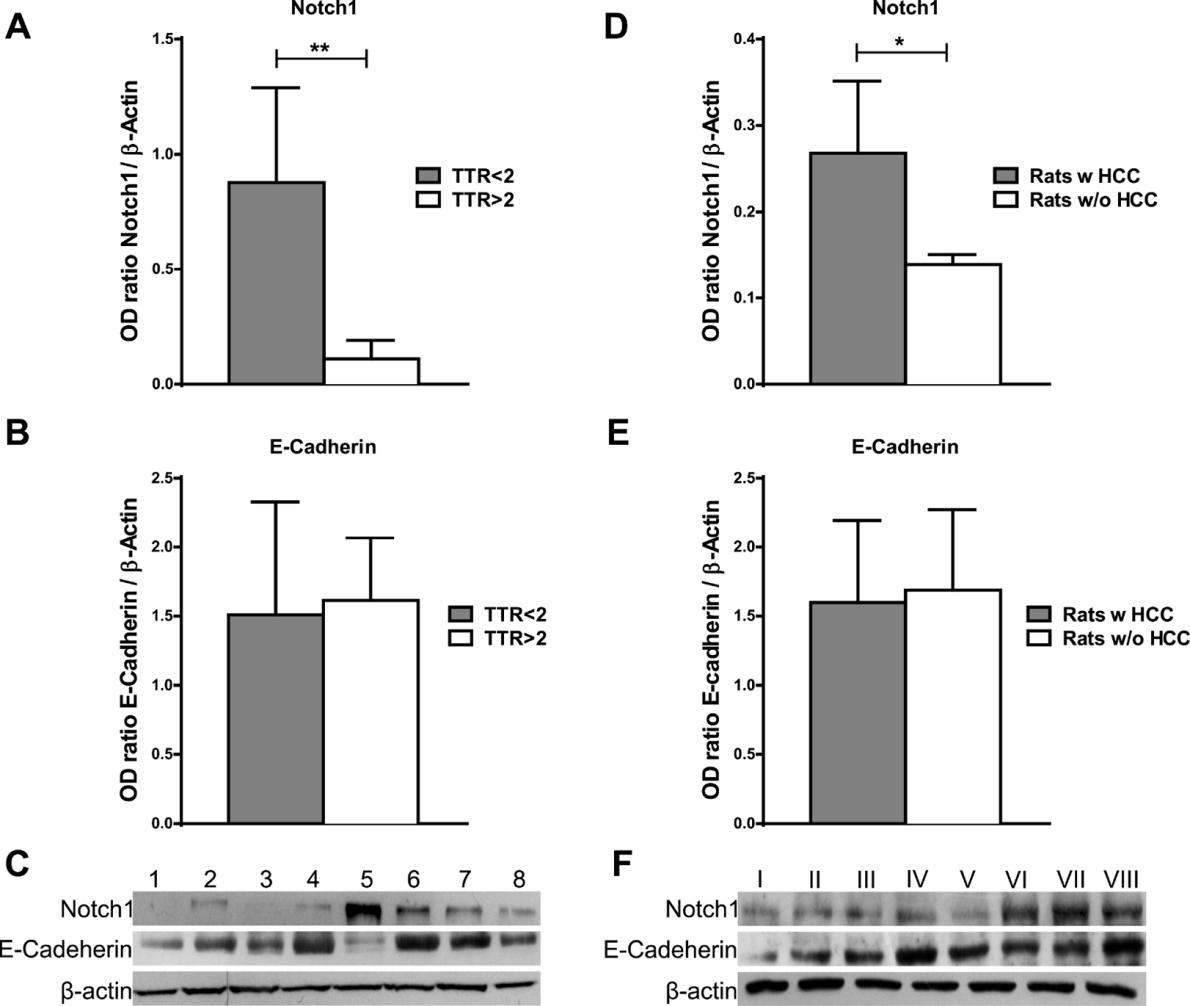
Notch1 and E-Cadherin expression in non-tumor liver tissue (**A**–**B**) Notch1 and E-Cadherin expression were evaluated by western blot in cirrhotic tissues surrounding HCC and a higher Notch1 expression was observed in cases with poor (< 2 years) vs. good (> 2 years) recurrence-free survival (TTR) **P < 0.01 (by two tailed student's *t* test). (**C**) Representative Notch1 and E-Cadherin expression in cirrhotic tissues with poor (< 2 years) (cases 5-8) vs. good (> 2 years) (cases 1-4) recurrence-free survival (**D**–**E**) Notch1 and E-Cadherin protein expressions were detected by western blot in liver of rats treated with DENA. Higher Notch1 expression was observed in non-tumor liver of rats that developed HCC (Rats w HCC) compared to those that did not developed (Rats w/o HCC). *P < 0.05 (by two tailed student's *t* test). (**F**) Representative Notch1 and E-Cadherin expressions in non-tumor liver of rats that developed HCC (cases V–VIII) compared to those that did not developed HCC (I–IV).

To further confirm the potential role of Notch1 in the increased risk of HCC recurrence, we analysed its expression in cirrhotic liver tissue surrounding HCC by western blot. Notch1 expression in cirrhotic tissues was higher in patients with low (< 2 years) vs. high (> 2 years) recurrence-free survival. On the contrary, no difference is observed in the expression of E-Cadherin between the two groups (Figure 5A–5B). These data suggest that Notch1 deserves attention as candidate for personalized therapies in the prevention of HCC recurrence after curative surgery.

Two distinct types of HCC recurrence are known: tumor grown from dissemination of the primary tumor and *de novo* tumor arising from “the field effect” in diseased liver [24]. The field effect is a carcinogenic microenvironment, which is assumed to cause accumulated genetic hits inducing cellular transformation [25].

To examine the possible involvement of Notch1 in generating this carcinogenic microenvironment we used the DENA model of liver carcinogenesis in rats [26](Supplementary Figure 4). Animals were treated with DENA for 8 weeks and then monitored by ultrasound for HCC development. Tumor's growth was observed in 88% of rats whereas HCC was not detected among the remaining rats. Since the overt tumors cannot be considered as early lesions, we assessed the contribution of Notch1 to the “field effect” of non-tumor liver tissue. Indeed, HCC outcome can be predicted using non-tumor liver tissue as previously reported [24]. Interestingly, western blot analysis showed higher Notch1 expression in the non-tumor liver of rats that developed HCC nodules compared to those that did not develop tumors (Figure 5D) suggesting a role of Notch1 in HCC development.

In agreement with the results in human cirrhosis, no differences were observed in the levels of E-Cadherin between the two groups (Figure 5E).

## DISCUSSION

In an effort to bring scientific knowledge from the bench to the bedside, several anti-Notch1 agents are under investigation [27] and it's of important interest to identify molecular biomarkers that can be used to predict tumor response or resistance to therapy. In this study, we used a proteomic approach comparing HepG2 and HepG2 Notch1 depleted cells to identify potential proteins biomarkers for predicting the efficacy of Notch1 inhibition. Quantitative proteomic analysis revealed changes in 89 proteins resulting from Notch1 silencing. Many of the identified proteins were initially classified as non-secreted, but further analysis revealed that they could use non-conventional secretion pathways. Similar findings were described for the secretome of other cancer cell lines [28]. They could be exosomal proteins, proteins cleaved from plasma membrane or derived from cellular breakage. We focused on proteins reported to be relevant in different pathways including cell adhesion, membrane organization and regulation of metabolic process. Due to keratins expression in some normal human tissues and in different diseases their serum levels might be modulated by health conditions in patients and were not validated [29]. After testing different proteins, Pai3, Thbs1 and sE-Cad show the ability to discriminate patients from controls whereas Icam5 and sE-Cad categorize early from advanced HCCs. In line with our results showing higher serum levels of sE-Cad in advanced HCC compared to early HCC, sE-Cad was associated to recurrence or extra-hepatic metastasis [30]. Thbs1 is considered an angiogenesis inhibitor associated to tumor invasion in melanoma, lung cancer, breast cancer and cholangiocarcinoma [31–33]. Accordingly, with our results, Thbs1 expression was reduced in HCC tissue compared with non-cancerous tissue [34].

Although validation using a larger sample set is required, our results provide evidence that Pai3, Thbs1, and sE-Cad could be promising serum biomarkers to predict response to Notch1 inhibitors, also based on the negative correlation between the expression of Notch1 in HCCs and serum Thbs1 levels evaluated by ELISA.

Recent evidences suggest that Notch1 is also associated with recurrence after surgery in human malignancies [35]. Surgery is the standard of care for patients with resectable HCC [36] however, long-term prognosis remains poor due to high recurrence rate associated with both dissemination of the primary HCC and de novo tumors arising from the ‘field effect’ in the diseased liver [37]. Herein we show that Notch1 promotes the invasion capabilities of HCC cells. To explore the potential mechanism involved in this process, we found out that down-regulated Notch1 cells reduce E-Cadherin protein expression. E-Cadherin has been identified as a tumor suppressor in different human cancer; however, several studies suggest that the role of E-Cadherin might be more complex [38]. E-Cadherin expression has been described to favor invasiveness and intra-hepatic metastases in some studies [39, 40] while others reported opposed results in HCC [9] suggesting that environmental factors are also involved in the routes of Hepatocellular carcinoma development. Herein we showed that siRNA mediated E-Cadherin knockdown reduces invasion capability of HepG2 cells. Interestingly, in down-regulated Notch1 cells, E-Cadherin over-expression reverted effect of Notch1 ablation on cellular invasion suggesting that both Notch1 and E-Cadherin contribute to invasion in HCC. In agreement with the *in vitro* data, TTR was shorter in patients displaying high levels of both Notch1 and E-Cadherin protein expression. On the contrary, the expression of E-Cadherin in cirrhosis cannot be considered as marker of early recurrence.

Finally to examine the possible involvement of Notch1 in generating a microenvironment responsible for the “the field effect” in diseased liver we used the DENA model of liver carcinogenesis in rats. We found out that Notch1 was expressed in the non-tumor liver of rats that developed HCC while it was not expressed in the liver of rats that did not develop hepatocellular carcinoma, suggesting that the lack of Notch1 protein expression could prevent HCC development. According to our observation, Notch1 increased during the hepatocarcinogenesis process induced by the methyl-deficient diet in rat liver [41]. Moreover, Notch1 signaling promotes liver carcinogenesis in a genetically engineered mouse model [42]. On the other hand, Huntzicker and coauthors described that Notch1 inhibition altered the relative proportion of tumor types, reducing HCC-like tumors but dramatically increasing cholangiocarcinoma-like tumors [16]. This is not surprising given that Notch signaling can play opposite biological effects that are highly context-specific in a time, gene-dose and cell type dependent manner [43].

In line with the results in human cirrhosis, the expression of E-Cadherin is similar in rats that developed HCC compared to those that did not develop cancer. Overall, our results suggest that the expression of E-Cadherin is a late event in the development and progression of cancer.

In conclusion, in this study we showed that Notch1 expression correlates with both tumor development and recurrence making Notch1 a good candidate for targeted therapies in HCC. Furthermore, Pai3, sE-Cad and Thbs1 can be used as serum markers of susceptibility to Notch1 inhibitors.

## MATERIALS AND METHODS

### Ethics statement

Investigation has been conducted in accordance with the ethical standards according to the Declaration of Helsinki and according to national and international guidelines and has been approved by the author's institutional review board.

### Cell lines and Notch1 knockdown by retroviral transduction of shRNAs

The human hepatocarcinoma cell lines HepG2, SNU398 and SNU449 were obtained from American Type Culture Collection (ATCC, Rockville, MD, USA) maintained in Media according to ATCC instructions. Notch1 knock down (KD) was obtained using short hairpin oligonucleotides (ShOligos) targeted to different Notch1 exons inserted into the pSuper.puro expression vector (OligoEngine, Seattle, WA) as previously described [13]. Since two Notch1 specific shRNAs were equally effective in our previous study [13] and here in the invasion assay, we performed the experiments by selecting a single shRNA (N1). Cells harbouring a pSuper.puro provirus expressing a GL2 luciferase specific shRNA were used as negative control (NC).

### Cell culture and conditioned media (CM)

HepG2 Notch1 silenced cells and GL2 control cells were cultured to 80% confluence in the medium as above described. Plated cells were washed 5 times with PBS and incubated in FBS-free media for 24 h at 37°C. At the end of the culture period, CM was removed and centrifuged at 1,500 rpm for 5 min. The supernatant was transferred into fresh tubes and a mixture of phosphatase and protease inhibitors was added. The CM was concentrated using acetone and protein concentration was determined using the Bio-Rad protein assay (Bio-Rad).

### Trypsin digestion

Twelve μg of protein extract was treated with 5 μl of ammonium bicarbonate (AMBIC) 100 mM, reduced with dithiothreitol (DTT, 10 mM, 1 μl in AMBIC 100 mM) at 56°C for 30′ and alkylated with 4-vinyl pyridine (55 mM, 1 μl in AMBIC 100 mM) at room temperature (RT) in the dark for 1 h. Alkylating agent excess was quenched with 10 μl DTT 10 mM which was left reacting at RT for 10′. The resulting protein mixtures were digested with TPCK-modified sequencing grade trypsin (final ratio of enzyme to substrate 1:50 w/w) at 37°C overnight. Samples were then acidified with 5% formic acid (FA) solution and dried in a vacuum evaporator. Peptides were resuspended in 30 ul of 1% FA/acetonitrile 98:2 solution.

### Mass spectrometry analysis and proteins identification

Analyses were performed by ESI-Q-TOF Accurate-Mass G6520AA (Agilent Technologies), controlled by MassHunter (v. B.02.00) and interfaced by a CHIP-cube to an Agilent 1200 nano-pump. Chromatographic separation was performed on a high capacity loading chip, with a 150 mm, 300Å, C18 column prior to a desalting step through a 500 nL trap column. Injected sample (2 μl, 0.8 μg) was loaded on the trap column with a 4 μl/min 0.1% FA:ACN 98:2 phase; after 3 min, the precolumn was switched in-line with the nano flow (400 nl/min, phase A: water:ACN:FA 96.9:3:0.1, phase B: ACN:water:FA 97:3:0,1). The peptides were eluted from the RP column through the following gradient: 3–45% B over a period of 75 minutes, 45–90% in 10 min, 90% B hold for 5 min, and back to 3% B in 8 min - a total of 110 min of runtime, including a 10 min post-run reconditioning step. Centroided MS scan spectra were acquired in positive mode in the range of 300 to 1700 Da with a 6Hz sampling; top 5 ions, preferring +2 and +3 species, were selected for MS/MS analysis, setting an active exclusion of the same precursor after 2 spectra over 0.15 minutes. Tandem mass spectra were recorded in the mass range 50 to 1700 Da with a sampling rate of 3 Hz. Collision energy was ramped with a slope of 3.6 and an offset of −3 V. Automatic QToF calibration was performed before each run. Each sample was run twice; analytical controls (a mix of baker's yeast enolase and bovine serum albumin tryptic digests) were run daily to monitor chromatographic performances.

MassHunter produced mzData.xml raw data that were searched against Swiss Prot database (SwissProt_57.15.fasta) using an in-house MASCOT Server (version 2.4, Matrix Science, UK) with the following settings: 30 ppm parent ion tolerance, 0.15 Da fragment ion tolerance, semi tryptic cleavage with two allowed missed cuts, pyridylethyl-cysteine and oxidized methionine as fixed. A concomitant search was performed with a database accounting for common non-human contaminants. MASCOT results were processed with Mascot Percolator, a semi-supervised machine learning algorithm for rescoring database search results (http://www.sanger.ac.uk/resources/software/mascotpercolator). A MASCOT ion score ≥ 50 for peptide identifications was required. Peptides reported by search engine were accepted only if they met the false discovery rate of *p* < 0.05.

### Label-free relative quantification

Exported re-scored MASCOT search results were cross-related to the corresponding MS1 profiles (mzXML format), converted from the vendor's raw data by trapper, http://sourceforge.net/projects/sashimi/files/trapper (Mass Hunter converter) by the quantitative software Ideal-Q [22], v. 1.024. Pooled search iMGN unique hits were manually validated and quantified on the moverz/RT 2D map. Peptide raw XIC data were exported and elaborated through the DanteR tool (https://omics.pnl.gov/software/danter) [23]. Briefly, peak areas were Log_2_ transformed, normalized (central tendency), then corresponding protein intensities were obtained from the peptides through an analysis of variance (*p*-value < 0.05, Log_2_0.5 < protein ratio > Log_2_2) and the inferred values were evaluated for statistical significance of differentially expressed proteins throughout the groups under study.

False discovery rate was estimated through a concatenated decoy database search and was lower than 1% in all the search results. Percolated hits were visualized by protein family grouping and exported as XML or CSV with MudPIT scoring, reporting only unique peptide hits and the highest ranked proteins of each family.

### Cell invasion assay

Cell invasion was assessed by Boyden blind-well chambers containing poly-vinyl-pyrrolidone–free polycarbonate filters, 8-μm pore size coated with Matrigel (Sigma, Saint Louis, MO, USA). Twenty-four hours after the transfection, 5.0 × 10^4^ HepG2, 3.0 × 10^4^ SNU449 and 3.5 × 10^4^ SNU398 cells were resuspended in serum-free medium and added to the upper chamber. A medium supplemented with 30% FBS was used as chemoattractant to the lower chamber. After 24 h and 48 h of incubations, non-invading cells were removed from the upper surface of the filter with cotton swabs. Invasive cells were fixed with 4% paraformaldehyde, stained with Giemsa (Sigma), and counted under a microscope.

### Wound healing assay

SNU398 and SNU449 cells were grown to confluence in regular medium, and then were maintained in serum-free medium for 48 h. The monolayers were scratched using a P200 pipette tip, rinsed and photographed (t0), (t1), (t2). Images are representatives of three different experiments. Scratched monolayers were fixed in cold methanol and incubated with Ki67 antibody (Dako) for the evaluation of cell proliferation in the wound area. Negative controls were obtained by omitting the primary antibody.

### Gelatin zymography

Gelatinolytic activity and quantity in conditioned media were analyzed by gelatin zymography as previously described [44].

### RNA analysis

Total cellular RNA was prepared using Trizol (InVitrogen, Paisley, Scotland) according to the manufacturer's instructions. One microgram of total RNA was reverse-transcribed using Superscript II (InVitrogen). Relative gene expressions were determined by semi-quantitative end-point PCR. PCR primers were reported in Supplementary Table 1.

### Transfections

HepG2 cells were seeded into 6 well plates and grown to ~40% confluence prior to Lipofectamine 2000 (InVitrogen) transfection with 40 nM of human E-Cadherin specific siRNAs or scrambled siRNAs (InVitrogen). Cells were harvested 24 h and 48 h after transfection and pellet was used for protein and extractions.

For plasmid transfection SNU398 cells were seeded into 6 well plates and transfected with 0,2 μg of pCMV6-XL4-CDH1 plasmid, containing the full length human CDH1 cDNA, or empty vector pCMV6-XL4 (OriGene Technologies, Rockville, MD) using Lipofectamine 2000. Analyses of proteins expression were performed 24 h post transfection.

### Immunocytochemistry

Cells were seeded on sterilized coverslips and fixed in 4% paraformaldeyde. Cells were then permeabilized in PBS containing 0.1% of saponin and incubated with normal goat serum at RT for 30 min. E-Cadherin and Notch1 proteins localization was assessed by using the same antibodies used in Western blot (Dako) followed by a HRP-rabbit EnVision system with diaminobenzidine (DAB) (Sigma) as chromogen. Cells were then counterstained with Mayer's hematoxylin and mounted with DPX (BDH Chemical, Poole, UK). Negative controls were obtained by omitting the primary antibody.

### Patients

HCC tissues from 38 patients (33 males and 5 females; median age: 70 y, range: 51–82 y) who underwent liver resection for HCC at the Department of Surgery of the University of Bologna, entered in the study. Tissue samples were collected during surgery and stored as previously described [45]. Informed consent was obtained from each patient. Histopathologic grading was scored according to Edmondson and Steiner's criteria (Edmondson HA, cancer). Exclusion criteria were a previous history of local or systemic treatments for HCC. In addition, to avoid cases in which aberrant methylation of E-Cadherin could represent a confusing factor to examine the relationship between Notch1 and E-Cadherin protein expression, HCC cases with low levels of E-CADHERIN mRNA were excluded from the analysis. Eighteen paraffin-embedded HCCs were obtained from the Addarii Institute of Oncology and Transplantation Pathology, S.Orsola-Malpighi Hospital and were used to assay Notch1 cellular localization.

### Immunohistochemistry

Notch1 expression was immunohistochemically assessed on 18 formalin-fixed, paraffin-embedded sections. Endogenous peroxidase was inhibited by incubating slides in 3% H_2_0_2_-methanol for 20 min at 4°C. For antigen retrieval, slides were immersed in pH 6.0 citrate buffer and boiled using a microwave oven. Negative controls were obtained by omitting the primary antibody. Immunoreactivity was revealed with the EnVision system (DAKO), and diaminobenzidine (DAB) as chromogen (Sigma). Slides were counterstained in Meyer's hematoxylin, coverslipped and examined by light microscopy. Staining of sections was assessed on 10 randomly selected fields at 40X by two independent observers (C.G, L.G.). Results represent the average of the percentage from ten 40X magnification fields.

### Serum analysis

Twenty one serum samples from patients (*n* = 7 cirrhosis, *n* = 7 HCC early and *n* = 7 HCC advanced) were obtained from Sant'Orsola-Malpighi Hospital. Written informed consent was obtained from all patients. Pools of healthy serum were also included in the study. None of these patients underwent surgery. Blood samples were left at RT for a minimum of 30 min (and maximum of 60 min) to allow clot formation, and then, centrifuged at 3000 g for 10 min at 4°C. The serum was aliquoted and stored at −80°C until use. Albumin was depleted according to the manufacturer's instruction of Proteo-Extract Albumin removal kit (Calbiochem, Germany). Albumin depleted serum was precipitated overnight in acetone, centrifuged at 18800 rcf for 20 min at 4°C and the pellets were dried in speed vacuum for 30 minutes at RT. Pellets were resospended in 8 M urea and a mixture of phosphatase and protease inhibitors.

### Thbs1 detection in human serum

Thbs1 was detected in the serum of 18 patients obtained from the Department of Surgery of the University of Bologna by using the human Thbs1 Elisa Kit (Biorbyt Ltd, Cambridge, United Kingdom) according to the manufacturer's instructions.

### Rats HCC induction

One hundred and twenty male Wistar rats were obtained from Harlan Italy (Udine, Italy) and were housed in an animal facility at Sant'Orsola-Malpighi Hospital (Bologna, Italy). Animals were maintained at a temperature of 20–22° and fed with a standard pellet diet ad libitum. The protocols of the experiments were approved by the Ethical Committee of the University of Bologna in accordance with European legislation. Hepatocellular carcinomas (HCCs) were induced by diethylnitrosamine (DENA) added in their drinking water for 8 weeks as previously described [46]. After this period, animals were monitored to check HCC's development by weekly ultrasound examination. One hundred and six rats developed liver nodules and were sacrificed as soon as the ultrasound showed the onset of HCC nodules. Tumor nodules and non-tumor liver tissue distant from the HCC nodules were collected and snap-frozen in liquid nitrogen or fixed in 10% formalin and paraffin embedded for histopathological analysis The remaining 14 rats were monitored for at least 2 months and no nodules were detected both at ultrasound examination and at sacrifice. Liver tissue was collected and snap-frozen in liquid nitrogen or fixed in 10% formalin and paraffin embedded for histopathological analysis. All 14 rats that did not develop HCC entered the study while among 106 rats that developed HCC, we chose 18 rats with less than 2 liver nodules each, of nodules less than 5 mm and without histopathological identifiable micro-lesions in the non-tumor liver distant from HCC.

### SDS-PAGE and Western blot analysis

Protein extraction and immunoblotting were performed as previously described [47]. Primary antibodies were as follows: anti-Notch1 (Clone A6 Novus Biologicals, Cambridge, UK), anti-Thbs1(sc-73158, Santa Cruz Biotechnology, Santa Cruz, CA, USA), anti-E-Cadherin (Clone NCH-38, Dako, Denmark) anti-mTor (2993, Cell Signaling Technology, Beverly, MA), anti-Icam5 (Abcam, Cambridge, UK), anti Pai3 (sc-99153, Santa Cruz Biotechnology), anti-Vimentin (Clone V9, Dako), anti Ck19 (Clone RCK108, Dako), anti-Ck18 (Clone DC10, Dako), anti-Mmp-9 (Clone 6-6B, Calbiochem, San Diego, USA), anti-Snail (sc-28199, Santa Cruz Biotechnology), anti-Ck8 (sc-52324, Santa Cruz Biotechnology), anti-Alpha-Sma (Clone 1A4, Sigma), anti-E-Cadherin (Clone 4A2, Cell Signaling) and anti-β-Actin monoclonal antibody (Clone AC-40). Immunoreactivities were revealed with the EnVision dextran polymer visualization system (Dako). Membranes were washed and autoradiographies were obtained using a chemiluminescence reaction (ECL reagents, Amersham). Digital images of autoradiographies were acquired with a scanner (Fluor-S MultiImager, Bio-Rad) and signals were acquired in the linear range of the scanner and quantified using specific densitometric software (QUANTITY-ONE, Bio-Rad) in absorbance units.

### Statistical analysis

After testing the distributions normality with the Shapiro-Wilk test, differences between groups were analysed using a double-sided Student *t*-test. Experimental data are expressed as the mean ± SE from three independent experiments. *T*-test was also used to search significant difference in Notch1 and E-Cadherin expression in human cirrhosis and in non-tumor rat liver tissues. Pearson's correlation was used to explore the relationships between Notch1 and E-Cadherin expression in human HCC tissues. Pearson's correlation was also used to explore the relationships between Notch1 expression in human HCC evaluated by immunohistochemistry and Thbs1 serum levels evaluated by ELISA. The Kaplan-Meyer survival analysis was used to compare patient's survival and time to recurrence (TTR) based on different Notch1 and E-Cadherin expression levels (the cut-off values were chosen on the basis of the median values) and statistical *p* value was generated by the Cox-Mantel log-rank test. Survival analyses were performed considering only cancer-related deaths whereas events related with other causes, including liver failure, were excluded.

*P*-values less than 0.05 were considered statistically significant. Statistical analyses were performed using SPSS version 19.0.

## SUPPLEMENTARY MATERIALS FIGURES AND TABLE


