# Supplementary material for: Molecular and proteomic insight into Notch1 characterization in hepatocellular carcinoma
Source: Oncotarget. 2016 May 6;7(26):39609–26. doi: 10.18632/oncotarget.9203 (PMC5129957; doi:10.18632/oncotarget.9203)
Supplement: Supplementary file 1 [file oncotarget-07-39609-s001.pdf]

Molecular and proteomic insight into Notch1 characterization in hepatocellular carcinoma

Supplementary Materials

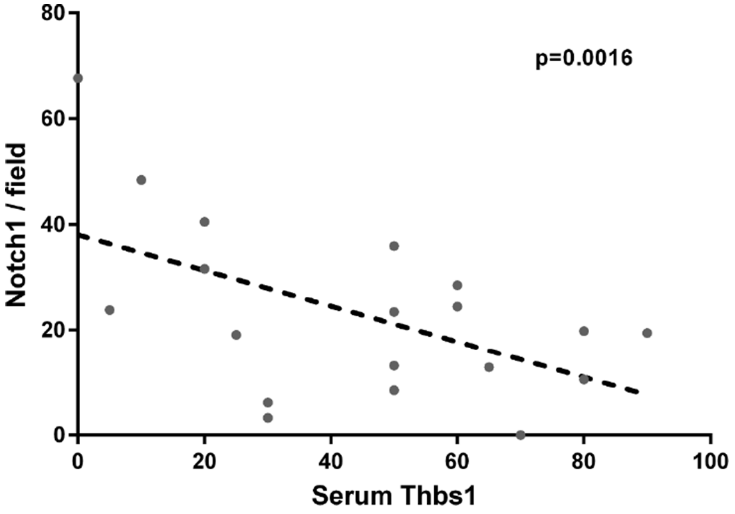

Supplementary Figure S1: Serum Thbs1 negatively correlates with Notch1 expression in human HCC. Scatter plot showing the negative correlation between serum Thbs1 evaluated by ELISA and Notch1 expression evaluated by Immunohistochemistry in human HCC. $P = 0.016$ (by two tailed student's t test).

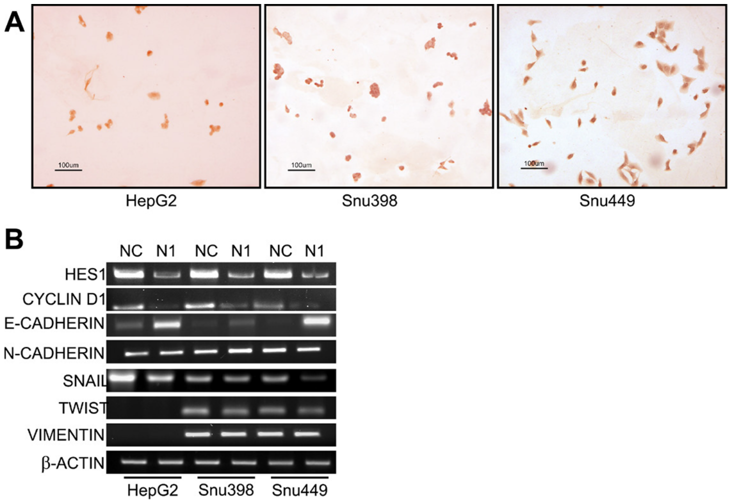

Supplementary Figure S2: Evaluation of Notch1 activity. (**A**) Expression and subcellular localization of the Notch1 intracellular domain (NICD), as detected by the immune-peroxidase method, of the HepG2, SNU398 and SNU449 cell lines. Positive staining was observed at the nuclear level. Nuclei were counterstained with hematoxylin. Original magnification 20X. (**B**) HES1, CYCLIN D1 E-CADHERIN, N-CADHERIN, SNAIL, TWIST and VIMENTIN expression was evaluated by RT-PCR in HepG2, SNU398 and SNU449 Notch1 silenced cells. β ACTIN was used as a reference control for mRNA levels NC: negative control shRNA; N1; Notch1 shRNA.

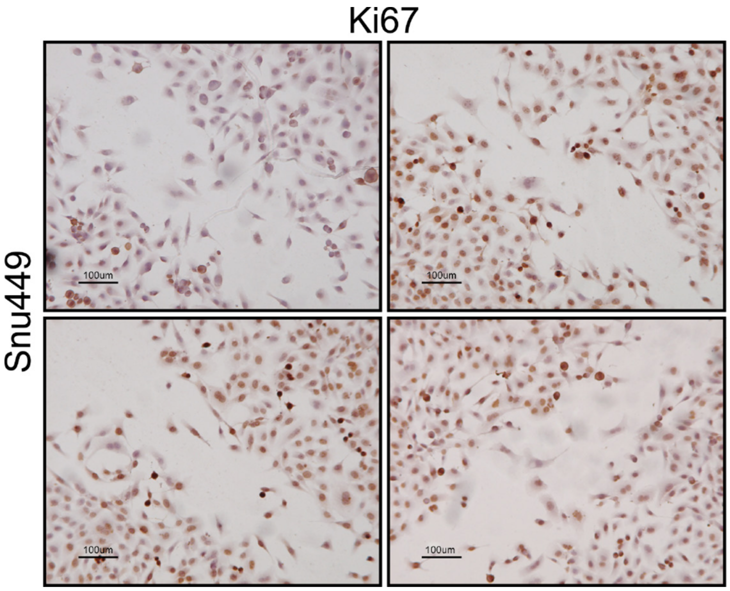

Supplementary Figure S3: Ki67 expression in the wound area. Confluent SNU449 cells were manually wounded by scraping the cell monolayer with a 200 ul pipette tip. The cell culture medium was replaced and Ki67 expression was assessed at 48 hours by the immune-peroxidase method. Nuclei were counterstained with hematoxylin. Original magnification 20X.

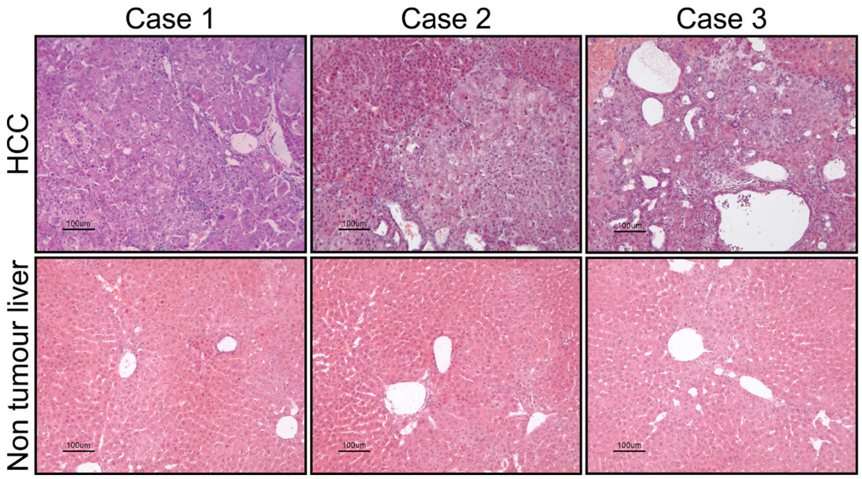

Supplementary Figure S4: Histological images of rat livers treated with DENA. Hematoxylin-eosin staining of the liver of three representative rats showing HCC and normal liver. Original magnification 20X.

Supplementary Table S1: Primer sequences for RT-PCR

Gene	Primers sequence (5′-3′)	Annealing T (°C)	Cycle n°	Product size (bp)*	Analysis
E-CAD F†<br>E-CAD R‡	TCCTGGGCAGAGTGAATTTTGA<br>GCGTGAGAAGAGAGTGTATGTGG	56	29	550	RT-PCR
SNAIL F<br>SNAIL R	ATCCAGAGTTTACCTTCCAGCAG<br>CCAGGACAGAGTCCCAGATG	65	33	145	RT-PCR
VIMENTIN F<br>VIMENTIN R	GAGAACTTTGCCGTTGAAGC<br>TCCAGCAGCTTCCTGTAGGT	57	29	150	RT-PCR
TWIST F<br>TWIST R	GAGACCTAGATGTCATTGTTTCCAG<br>CACGCCCTGTTTCTTTGAAT	65	33	104	RT-PCR
N-CAD F<br>N-CAD R	GACAATGCCCCTCAAGTGTT<br>CCATTAAGCCGAGTGATGGT	58	29	179	RT-PCR
β ACTIN F<br>β ACTIN R	GAGGCACTCTTCCAGCCTTC<br>GGATGTCCACGTCACACTTC	55	26	189	RT-PCR

*bp, base pairs.
†F, forward.
‡R, reverse.
